# Effectiveness of lipid‐based nutrient supplementation during the first 1000 days of life for early childhood development: A community‐based trial from Pakistan

**DOI:** 10.1111/mcn.13727

**Published:** 2024-09-24

**Authors:** Ayesha Imtiaz, Zia ul Haq, Suhail A. R. Doi, Sheraz Fazid, Muhammad Naseem Khan

**Affiliations:** ^1^ Institute of Public Health & Social Sciences Khyber Medical University Peshawar Pakistan; ^2^ Institute of Health & Wellbeing University of Glasgow Glasgow UK; ^3^ Department of Population Medicine College of Medicine, QU Health, Qatar University Doha Qatar

**Keywords:** caregiver, child development, cognition, health education, nutrient supplement, Pakistan, pregnant women

## Abstract

A community‐based, cluster non‐randomized controlled trial was conducted in Kurram district, Pakistan between January 2018 to December 2020. Age‐appropriate lipid‐based nutrient supplements and health education (sessions conducted in the households) were given to pregnant women and their born children (6–23 months) in the intervention arm (*n* = 40 clusters) versus health education only in the control arm (*n* = 40 clusters) to evaluate its effect on child development. The first and second developmental assessments were completed at ~24 months (*n* = 689) and ~32 months (*n* = 608), respectively, using the Caregiver‐Reported Early Development Instrument Long form. The overall and domain‐specific (motor, language, cognitive and socio‐emotional) scores were computed with higher scores indicating better child development. Higher development scores, including overall (*β* = 0.40, 95% confidence interval [CI]: 0.14, 0.65; *p* = 0.002), cognitive (*β* = 0.27, 95% CI: 0.10, 0.45; *p* = 0.002), motor (*β* = 0.39, 95% CI: 0.22, 0.56; *p* < 0.001) and language (*β* = 0.33, 95% CI: 0.14, 0.51; *p* = 0.001) were reported for children who received the intervention compared to the control arm at first developmental assessment. However, the effect was not sustained after the discontinuation of the intervention. The LNS received by the mothers (during pregnancy and first 6 months after delivery) and by children during 6–23 months of age was beneficial for the children. The trial is registered in the International Standard Randomised Controlled Trial Number Registry (ID: ISRCTN94319790) on December 11, 2017.

## Key messages

- In Pakistan, 54% of children under five are suggested to be at risk of poor development.
- Optimal brain development requires adequate nutrition during pregnancy and the first few years of life.
- The lipid‐based nutrient supplement received by the mothers (during pregnancy and the first 6 months after delivery) and by their children during 6–23 months of age improved the child's development.
- Interventions for longer duration or long‐term follow‐up studies are required to observe the sustained effect of intervention.

## 1 INTRODUCTION

Early childhood development (ECD) is widely recognized as an important predictor of educational achievement, lifelong health and well‐being, economic productivity and strong communities (Centre on the Developing Child, n.d.). For development to be sustainable, young children must be given the chance to reach their maximum developmental potential (World Health Organization [WHO], 2020). ECD is therefore included in the sustainable development goals, under Target 4.2 (McCoy, Waldman, et al., 2018) and many other global agendas (Richter et al., 2019). The early years of life hold critical importance in the child's development. Early childhood is a period when children develop basic developmental skills (McCoy, Waldman, et al., 2018), including physical, sensorimotor, social, emotional, language and cognitive (Pérez‐Escamilla & Moran, 2017). The early years are especially important, being the time when the brain develops rapidly, with the most rapid development occurring in the first 3 years of life (WHO, 2020). In addition, the capacity of the brain to reorganize and adjust to the environment (plasticity) is maximum during the early years of life and decreases with age (Centre on the Developing Child, n.d.; Fernald et al., 2017).

However, there is little public understanding of the importance of a child's early years of life and negligible public demand for policies, programmes and funding (UNICEF n.d.). According to an estimate, 249 million children under the age of 5 (43%) living in low‐ and middle‐income countries (LMICs) cannot develop to their full potential due to poverty and stunting (Richter et al., 2019). The situation is even worse in Pakistan, where 54% of children under 5 are suggested to be at risk of poor development (NURTURING‐CARE.ORG, 2021). Neglect and inaction have a high price and long‐term implications for the health, well‐being and earning potential as adults (UNICEF, n.d.). Poverty, malnutrition, stress, violence and insecurity, gender inequities, environmental toxins, and maternal poor mental health are among the major risk factors for suboptimal child development (Richter et al., 2019; WHO, UNICEF, World Bank Group, 2018). Children's ability to achieve their full developmental potential can be improved by preventing exposure to risks or taking steps to reduce their impact on development (Fernald et al., 2017).

Optimal brain development requires adequate nutrition during pregnancy and the first few years of life (Taylor et al., 2017). The first 1000 days of life (the period from conception to the age of 24 months after birth) provides a window of opportunity to improve the child's survival, growth and development (Matsungo et al., 2017). Various interventions are recommended to promote child growth and development (Das et al., 2019). One of the nutritional interventions advocated to improve the child's growth and development is lipid‐based nutrient supplements (LNS). LNS provides energy, protein and essential fatty acids in addition to a variety of vitamins and minerals. LNS is currently being used in programmes targeting pregnant women and children 6–23 months of age in LMICs with the expectation to improve birth outcomes as well as child growth and development (Das et al., 2018). Studies assessing the impact of LNS on development in infants and young children have given mixed results (Das et al., 2019). The most recent evidence supports the positive effect of LNS on ECD; however, in the majority of studies the LNS was given to children 6–24 months of age (Prado et al., 2021). Scientific evidence related to the effect of a nutrition intervention during the first 1000 days of life (starting in pregnancy, first 6 months postpartum and then till the age of 2 years) on ECD is limited in Pakistan and is lacking for LNS. In addition, few studies have assessed the effectiveness of nutrient supplementation programmes on young child development using the existing government health services (Larson et al., 2018; Yousafzai et al., 2014).

This study was conducted to assess the effectiveness of LNS given during the first 1000 days of life for the development of children under 3 years of age in motor, cognitive, language and socio‐emotional domains compared to those only receiving health education in the control group, in the Kurram district. The conceptual framework underlying our research was the ‘Nurturing Care Framework’, developed for promoting ECD in May 2018. Nutrition is one of the five strategic areas outlined in the framework, suggested to be effective in improving ECD in both developed and underdeveloped countries (WHO, UNICEF, World Bank Group, 2018).

## 2 METHODS

### 2.1 Study design and setting

This study is part of a community‐based‐cluster non‐randomized controlled trial with parallel arm design conducted in Upper Kurram between January 2018 and December 2020. The trial is registered in the International Standard Randomised Controlled Trial Number Registry (ID: ISRCTN94319790) on December 11, 2017. The study received ethical approval by the Ethics Review Board (ERB), and is reported as per the CONSORT statement for cluster randomized trials (Campbell et al., 2012).

### 2.2 Study participants and eligibility criteria

Households in the catchment area of selected health facilities were included in the trial. Pregnant women who were residents, between 15 and 49 years of age, and in their first trimester (preferably first month of pregnancy) were included in the study. Children born to these pregnant women were eligible for enrollment for developmental assessment. Children with congenital malformations identified at birth, and severe developmental impairments such as cerebral palsy were excluded from the study. Children for which LNS was contraindicated (malabsorption/metabolic disorder) or unable to take LNS (e.g., cleft palate) were also excluded from the study.

### 2.3 Sample size

The design effect (DE) was given as [(n−1)ρ+1] where *n* = 15 was the chosen cluster size and ρ=0.05 (Kirkwood et al., 2023) was the estimated intracluster corelation coefficient, which is the proportion of outcome variance that is between clusters (*k*) rather than between individuals within clusters. We estimated the DE to be 1.7. We estimated the effective sample size required for an individually allocated trial to have the same power (*β* = 0.2) and precision (*⍺* = 0.05), as this two‐stage cluster trial given the effect size (MD = 0.21) (Yousafzai et al., 2016) and its estimated variance (SD = 1) to be 705 children (Schoenfeld, n.d). We then proceeded to multiply this by 1.7 and divide by 15 to determine the total number of clusters required for the trial which was determined to be 80. The data analysis therefore proceeded as an individually allocated trial.

### 2.4 Recruitment and allocation of participants

A two‐stage cluster sampling technique was used for the selection of study participants. A total of 80 clusters were selected randomly from the eligible clusters (*n* = 122). Households (~100–150) officially assigned to the Lady Health Workers (LHWs) were declared as intervention clusters and around a similar number of households (~100–150) with no official LHW (the district polio microplanning data were used for selection of households in the control clusters) declared as control clusters (WHO, 2018). For each treatment cluster, one control cluster was formed in a non‐randomized fashion, because randomization was not possible by design as the supplements were distributed to all the LHW‐covered areas. A cluster design was used to avoid contamination between the intervention and control participants. Further details about the methodology are published elsewhere (Fazid et al., 2024).

In intervention clusters, LHWs officially record each pregnant woman in their allocated households. All those pregnant women were invited to participate in the trial. Additional active searches were done to ensure each pregnant woman was enrolled. For the control clusters, an active search was done and each household visited to identify eligible pregnant women. This usually resulted in about 15 pregnant women per cluster. The woman's pregnancy status was verified by asking about the date of her last menstrual period, and possible pregnancy confirmed by a doctor. The recruited pregnant women were then followed on a monthly basis until delivery or termination of pregnancy and then as lactating women for the first 6 months after delivery while exclusively breastfeeding their children. Children born to pregnant women enrolled in the trial were included in the study and were followed until the final assessment.

Masking of the study participants, LHWs, and data collectors was not possible due to the nature of the intervention. Data collectors accompanied the LHWs during home visits and were present at the time of food distribution. The food supplements for pregnant/lactating women and children were in a different colour sachet, evident to participants and data collectors.

### 2.5 Intervention

The intervention was the supplementation of pregnant women and their children (6–23 months of age) with age‐appropriate ‘lipid‐based nutrient supplements (LNS)’ combined with health education. The intervention was delivered in selected clusters through the LHWs for a period of ~32 months, integrated within existing services through home visits (Yousafzai et al., 2014). LNS for the trial were locally manufactured by an international organization following established international standards and guidelines for quality and food safety (WHO, UNICEF, World Bank Group, 2012). The nutrient content of the LNS sachet (Maamta & Wawa mum) is given in Supporting Information S1: Table A1 **.**

In the intervention arm, LHWs were assigned the task of distributing the LNS to the study participants in addition to their routine official duty in their assigned catchment areas. The LHWs got the monthly ration of LNS from the health facilities for distribution during routine home visits to the research participants (*n* = 30 sachets per participant per month). Along with the delivery of LNS, LHWs in both the arms gave health education at each monthly home visit. The health education components included health, hygiene and basic nutrition education. The study participants were provided education on the following (i) regular antenatal visits for pregnant women to have healthy and nutritious food for themselves and their children; (ii) optimal infant and young child feeding practices as per recommended guidelines (WHO, 2021); (iii) maternal and children vaccination and their benefits; and (iv) personal and environmental hygiene. LHWs also educated mothers on the use of the nutrient supplements, and benefits and avoid sharing with other members of the household.

### 2.6 Outcome and data collection tools

The primary outcome was the mean score in overall and domain‐specific (motor, cognitive, language, socio‐emotional) child development assessed through the Caregiver‐Reported Early Development Instrument (CREDI) Long Form. CREDI is designed for the developmental assessment of children from birth to 3 years of age (McCoy et al., 2018a). CREDI is a validated population‐level development assessment tool (Altafim et al., 2020; Li et al., 2020; McCoy, Waldman, et al., 2018; Waldman et al., 2021) exhibiting adequate internal consistency, test–retest reliability, concurrent validity and sufficient reliability for population‐level measurement, when a multidimensional item factor analysis framework was employed (Waldman et al., 2021). CREDI Long Form is designed to assess the child's development through the achievement of developmental milestones (motor, language, cognitive and socio‐emotional) as well as the measurement of children's behaviours. It has 117 items in total; 108 items measure developmental milestones, having items for motor (*n* = 35), language (*n* = 22), cognitive (*n* = 10) and socio‐emotional (*n* = 15) skills, while 26 items measure more than one domain. An additional nine items assess mental health. Administration time was ~15 min. For all CREDI items, there were three response options: ‘Yes’ and ‘No’ represented by numeric values of ‘1’ and ‘0’, respectively. The ‘Don't Know’ response was treated as a missing value. For the scoring app package to generate a score, at least five items should have been responded to with ‘1’ or ‘0’ (McCoy et al., 2018b; Seiden et al., 2021). Overall and domain‐specific (motor, cognitive, language and social‐emotional) scaled scores were generated by the CREDI scoring app (1.0) software. An increase in average score with age indicates developmental progression (Seiden et al., 2021).

CREDI was translated into Pashto (the local language) with back translation done, with the help of linguistic experts. The questionnaire was shared with data collectors (residents) and piloted in Kurram and required changes were made as per feedback. The questionnaire required minimal changes including the replacement of some Pashto words spoken in the local language, for example, words used for ‘small toy’, ‘whisper’, ‘lying on the stomach’, ‘help’, ‘play on his or her own’ ‘forward’, and so forth. For the study, a 3‐day training session was arranged at the study site to train data collectors as per CREDI training guidelines (McCoy et al., 2018a).

Data were collected on household characteristics, demographic characteristics of the target population, including women's age, marital status, living structure, and years of schooling, and occupation of the women and their husbands, socioeconomic status and household food insecurity (HFI). Maternal obstetrics data were recorded, including information on pregnancy outcomes which included the child's date of birth, gender, birth weight, and gestational age. Data on infant and young child feeding practices were collected, including as per recommended guidelines (WHO, 2021).

Anthropometric data of pregnant women and children were collected including length/height (cm), weight (kg) and midupper arm circumference (MUAC). Weight was measured using digital weighing scale (SECA) and length/height was measured using SECA height board. Weighing was done with light clothing and weighed to the nearest 0.1 kg. Length/height was measured to the nearest 0.1 cm, following the standard procedures (WHO, 2008). MUAC was measured using flexible insertion tape. Three measurements were recorded for each anthropometric variable and their average was taken.

### 2.7 Data collection

Data collection of the participants was done through a designated team of 20 team leaders (4 clusters each). The team was thoroughly trained for a period of 1 week on the baseline and followup data collection. Potentially eligible participants were approached and invited to enrol. Written informed consent was obtained at baseline assessments after the information sheet and full trial informed consent form was shared and discussed with participants.

After enrollment, a cross‐sectional survey was conducted in the selected intervention and control clusters to collect information on baseline characteristics of the participants using the structured questionnaire. The developmental assessment was done in batches; therefore, the ages of the children varied at the time of the first and second developmental assessments. The first assessment of these children was from August to October 2020 (mean age: ~24 months) and the second developmental assessment was done from May to July 2021 after the enrollment (mean age: ~32 months). There was some delay in the assessment due to the COVID‐19 pandemic. This assessment was done using the CREDI Long Form.

To ensure accurate data collection, the project assistant and Principal Investigator (PI) double‐checked the collected data for any missing or inconsistent data. In case of any errors or missing data, the data collectors were contacted and inquired about and asked for a correction. Participants’ data were stored in locked cabinets with the PI and then transferred to password‐protected computers. In addition, data for each participant were assigned a unique identifier to maintain confidentiality and data integrity. Morbidity data were collected every month as part of the monthly data collection during the follow‐up, and any harm (nausea, vomiting, diarrhoea, allergic reactions, etc.) that could come from participation in the trial was recorded.

### 2.8 Data analysis

Descriptive statistics including the mean (SD) for continuous variables and frequency/percentages for categorical variables to analyse the baseline characteristics of the participants. Data were checked for normality (mean/median distribution, histogram). Dependent and independent variables were examined for outliers (box plots) which were removed before analysis where required. Independent sample *t* test (for continuous variables) and chi square test (categorical variables) were performed to compare the intervention and control arms in terms of participant characteristics and reported as *p* value. A *p* value of <0.05 was taken as significant.

In the current study, there was significant loss of pregnant women and children over time, the loss to follow‐up was adjusted for by running appropriate weighted regression with weights derived from the logistic regression model. First, propensity scores were computed by using logistic regression, where the outcome variable was an indicator of participation (1 for participating, 0 for not participating) and the predictors were baseline explanatory variables. This yielded a predicted probability for each woman to participate at each time point based on her characteristics (Rattan et al., 2013).

An inverse probability linear regression analysis controlling for confounding factors was used to assess the effect of the intervention on child development. There was no need to adjust for clustering (see sample size calculations). A minimally sufficient set of confounding covariates was determined using a directed acyclic graph (Supporting Information S1: Figure A1). There were no highly correlated variables in the data and goodness of fit and goodness of link were assessed for all regression models and reported. Exact *p* values were reported throughout. All data were analysed using Stata version 17.

### 2.9 Ethical statement

The study received ethical approval by the Ethics Review Board (ERB) of a Medical University. Research was conducted in accordance with the ethical guidelines of the Declaration of Helsinki.

## 3 RESULTS

**Figure 1:**
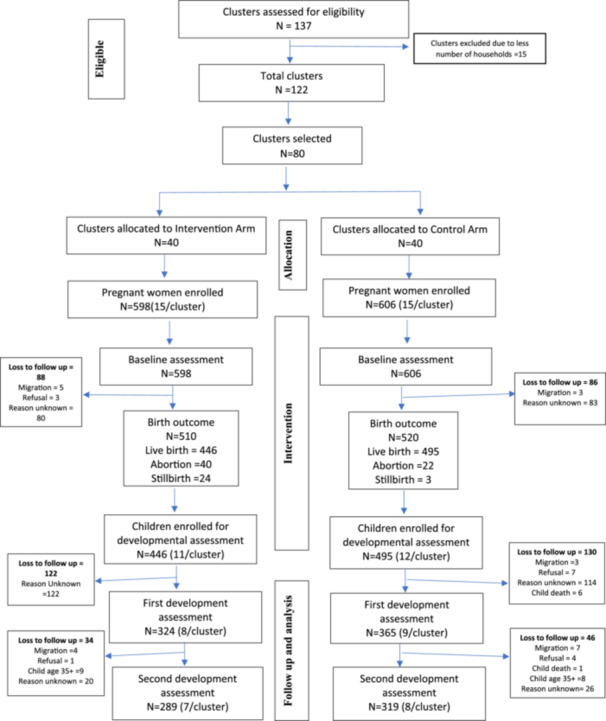
Flow diagram of the Trial.

There was a total sample of *n* = 1204 pregnant women, with *n* = 598 in the intervention arm and *n* = 606 in the control arm. The study flow diagram is given (Figure 1). Intervention and control arms were found to be comparable in terms of maternal characteristics (age, height, weight, dietary diversity), household characteristics (living structure, household members, household latrines) and child characteristics (age, and mean duration of breastfeeding) (Table 1). Some of the variables were not similar across the arms like maternal MUAC, poverty score, HFI score, mean weight and height of child among others. However, these differences were quite minimal. Almost all (99%) women were married and 97% were housewives. Households in the intervention arm were more food secure compared to the control arm. More women in the control arm took iron supplements during pregnancy. More participants in the control arm took measures to make drinking water safe. There were a total of 941 live births born to pregnant women in both arms. A higher proportion of male children was born to women in the control arm.

**Table 1:** Baseline characteristics of participants by study arm (*n* = 1204).

	Intervention	Control	
	*n* = 598 (49.7%)^a^	*n* = 606 (50.3%)^a^	
Participant characteristics	Mean (SD),^b^ *n* (%)	Mean (SD),^b^ *n* (%)	*p* Value
Socio‐demographic characteristics			
Maternal age (years)^a^	27.9 (5.9)	27.9 (5.8)	0.88
Mother years of schooling^a^	2.8 (4.5)	2.3 (4.2)	0.04
Father years of schooling^a^	7.2 (4.9)	7.1 (4.9)	0.62
Maternal Height (cm)^a^	157.7 (5.6)	157.5 (5.7)	0.40
Maternal Weight (kg)^a^	60.9 (11.0)	61.6 (11.3)	0.27
Maternal MUAC^a^	26.5 (3.3)	25.9 (3.2)	0.006
Poverty score^a^	42.6 (14.5)	40.4 (14.9)	0.009
HFI score^a^	3.9 (5.2)	4.6 (5.2)	0.027
Household members^a^	14.4 (8.8)	14.6 (9.5)	0.79
Household latrines^a^	1.50 (1.1)	1.62 (1.6)	0.38
Dietary diversity^b^			
Yes	71 (11.77)	88 (14.52)	0.18
No	527 (88.13)	518 (85.48)	
Living structure^b^			
Nuclear/Single	94 (15.72)	105 (17.33)	0.45
Joint/extended	504 (84.28)	501 (82.67)	
Measures for safe drinking water^b^			
Yes	40 (6.69)	146 (24.09)	<0.001
No	558 (93.31)	460 (75.91)	
Iron supplementation during pregnancy^b^			
Yes	237 (39.63)	309 (50.99)	<0.001
No	361 (60.37)	297 (49.01)	
Child characteristics			
Gender^b^			
Male	219 (49.10)	307 (62.02)	<0.001
Female	227 (50.90)	188 (37.98)	
Children age at first assessment (months)^a^	24.5 (2.6)	24.7 (2.6)	0.38
Children age at second assessment (months)^a^	32.5 (2.5)	32.9 (2.5)	0.08
Mean Weight (kg)^a^	13.39 (1.38)	14.12 (2.07)	<0.001
Mean Height (cm)^a^	89.9 (4.53)	88.76 (6.41)	0.005
Mean MUAC (cm)^a^	15.27 (1.17)	15.87 (1.19)	<0.001
Birth weight (kg)^a^	3.11 (0.41)	3.07 (0.43)	0.14
Mean duration of breastfeeding (months)^a^	19.07 (7.25)	20.00 (6.99)	0.11
Mean month of starting complementary feeding^a^	7.72 (1.65)	7.18 (0.78)	<0.001

Abbreviations: cm, centimetre; HFI, Household Food Insecurity; kg, Kilogram; MUAC, midupper arm circumference.

^a^ Mean (SD).

^b^ *n* (%).

Mean development scaled score in intervention and control arms at first and second developmental assessments are given in Table 2 **.** At the first developmental assessment, after controlling for covariates, the estimated effect of the intervention suggested an increase in child development scores, with strong evidence against the model hypothesis at this sample size (Table 3). The overall development score was positive (*β* = 0.40, 95% CI: 0.14, 0.65; *p* = 0.002) in the intervention arm compared to the control arm. The LNS received by the mothers (during pregnancy and first 6 months after delivery) and by children during 6–23 months of age was therefore beneficial for the children. Similarly the domain‐specific scores were also positive in the intervention arm, including cognitive (*β* = 0.27, 95% CI: 0.10, 0.45; *p* = 0.002), motor (*β* = 0.39, 95% CI: 0.22, 0.56; *p* ≤ 0.001), and language (*β* = 0.33, 95% CI: 0.14, 0.51; *p* = 0.001) all with strong evidence against the model hypothesis at this sample size, except for socio‐emotional development (*β* = 0.12, 95% CI: −0.04, 0.29; *p* = 0.148).

**Table 2:** Child development scores in intervention and control arms at first and second developmental assessment.

	Cognitive	Language	Motor	Socioemotional	Overall
Study arm	Mean (SD)	Mean (SD)	Mean (SD)	Mean (SD)	Mean (SD)
First developmental assessment					
Intervention	50.40 (1.03)	51.26 (1.21)	50.57 (1.07)	50.94 (1.09)	51.21 (1.6)
Control	50.15 (1.09)	50.98 (1.22)	50.14 (1.21)	50.69 (1.17)	50.74 (1.73)
Total	50.27 (1.08)	51.11 (1.22)	50.35 (1.17)	50.81 (1.14)	50.96 (1.70)
Second developmental assessment					
Intervention	51.45 (1.01)	52.41 (0.97)	51.97 (1.14)	52.22 (1.08)	52.79 (1.18)
Control	51.65 (1.05)	52.85 (1.20)	52.15 (1.26)	52.44 (1.03)	52.18 (1.30)
Total	51.55 (1.03)	52.63 (1.12)	52.06 (1.21)	52.4 (1.06)	52.99 (1.26)

**Table 3:** Estimated intervention effects for overall and domain specific development, compared between intervention and control arms at the first development assessment (*n* = 689).

Child development scores	*B* coef. (95% CI)	*p* Value	*R* ^2^	Link test (_hatsq)
Overall				
Intervention	0.40 (0.14, 0.65)	0.002**	0.11	0.253
Cognitive				
Intervention	0.27 (0.10, 0.45)	0.002**	0.11	0.078
Motor				
Intervention	0.39 (0.22, 0.56)	<0.001**	0.13	0.316
Language				
Intervention	0.33 (0.14, 0.51)	0.001**	0.19	0.091
Socioemotional				
Intervention	0.12 (−0.04, 0.29)	0.148	0.15	0.016

*Note*: Models adjusted for child age, gender, poverty score, household food insecurity score, mother education, mother age, father education, living structure. Comparing intervention with the control group.

** *p* < 0.01.

Estimated intervention effects for overall and domain‐specific development scores, compared between intervention and control arms at the second development assessment, are given in Table 4. The estimated effect of the intervention was negative, overall (*β* = −0.35, 95% CI: −0.54, −0.16; *p* < 0.001), cognitive (*β* = −0.24, 95% CI: −0.41, −0.07; *p* = 0.006), motor (*β* = −0.25, 95% CI: −0.44, −0.05; *p* = 0.013), language (*β* = −0.42, 95% CI: −0.59, −0.24; *p* < 0.001) and socioemotional (*β* = −0.23, 95% CI: −0.40, −0.056; *p* = 0.009) this time with strong evidence against the model hypothesis at this sample size. These results indicate that the effect of the intervention was not sustained on child development after the intervention stopped. The unweighted analysis was also done. Results are comparable to those of weighted analysis both at first and second developmental assessments indicating that attrition did not affect the results.

**Table 4:** Estimated intervention effects for overall and domain‐specific development, compared between intervention and control arms at second development assessment (*n* = 608).

Child development scores	*B* Coef. (95% CI)	*p* Value	*R* ^2^	Link test (_hatsq)
Overall				
Intervention	−0.35 (−0.54, −0.16)	<0.001**	0.15	0.579
Cognitive				
Intervention	−0.24 (−0.41, −0.07)	0.006**	0.14	0.295
Motor				
Intervention	−0.25 (−0.44, −0.05)	0.013*	0.14	0.533
Language				
Intervention	−0.42 (−0.59, −0.24)	<0.001**	0.17	0.374
Socioemotional				
Intervention	−0.23 (−0.40, −0.056)	0.009**	0.15	0.068

*Note*: Models adjusted for child age, gender, poverty score, household food insecurity score, mother education, mother age, father education, living structure. Comparing intervention with control group.

* *p* < 0.05

** *p* < 0.01.

## 4 DISCUSSION

The cluster non‐randomized controlled trial conducted in Kurram district in which LNS was given to women during pregnancy and the first 6 months after delivery and to children from 6 to 24 months of age showed positive effect of the intervention on child development scores, both overall and specific domains (motor, language, cognitive and socio‐emotional). However, the effect of the intervention was not sustained after the intervention was stopped.

The positive effect of intervention on overall and domain‐specific child development scores at 24 months of age in our study is supported by two recent systematic reviews and meta‐analysis which analysed the effect of Small Quantity LNS (SQ‐LNS) on various developmental outcomes among children. Tam et al. (2020) generated pooled estimate of studies (*n* > 3600 children), showing positive effect of LNS on mean language score (effect size: 0.13 SD), motor score (effect size: 0.13 SD), socio‐emotional score (effect size: 0.12 SD), with no effect on executive function. Further evidence is provided by Prado et al. through reporting Individual Participant Data (IPD) meta‐analysis (Prado et al., 2021), including 13 trials and a large sample of children (*n* = 30,024). Meta‐analysis showed an increase in mean language scores (MD: 0.07), socio‐emotional scores (MD: 0.08) and motor scores (MD: 0.08)**.** Both of these reviews included the studies in which LNS was given to children from 6 to 24 months of age with no study included where the supplements were started in pregnancy and then followed after delivery till the age of 2 years.

Randomized controlled trials investigating the effect of LNS given to mothers during pregnancy, lactation, and to children from 6 to 24 months of age have reported inconsistent findings (Addo et al., 2020). In two efficacy trials, one in Ghana (Prado, Adu‐Afarwuah, et al., 2016) and one in Malawi (Prado, Maleta, et al., 2016), SQ‐LNS were given both to mothers prenatal and postnatal and to children from 6 to 18 months of age. Although there were favourable effects on motor development at age 12 months, there were no discernible effects on executive function, motor, cognitive, or socio‐emotional development at age 18 months in those studies (Prado, Adu‐Afarwuah, et al., 2016; Prado, Maleta, et al., 2016). A cluster‐randomized trial in Bangladesh found a significant positive effect of SQ‐LNS on child motor and language development during the first 24 months of life. However, no effects were observed on personal‐social development, or executive function (Matias, Mridha, et al., 2017). These findings may suggest that the effect of the nutrition intervention may be more evident at the age of 2 years and beyond (Larson et al., 2018; Matias, Mridha, et al., 2017; Matias, Vargas‐Vásquez, et al., 2017) and might need longer follow up (Tofail et al., 2018).

The evidence suggests that LNS is effective for prevention of malnutrition, and improved growth and development of children in resource‐poor settings, where nutrition gaps exist in the diet of children. SQ‐LNS is reported to support motor, socio‐emotional and cognitive development of children equivalent to 1–5 IQ points depending on the nutritional status of children (UNICEF, 2023). Benefits of LNS are reported to be more among study population with higher rates of stunting, lower socioeconomic status and children with low maternal education (Prado et al., 2021). Similarly, a greater effect of postnatal LNS on motor development was observed in children living in low‐stimulating home environments in Bangladesh (Matias, Mridha, et al., 2017), Burkina Faso (Prado et al., 2015) and Bihar, India (Larson et al., 2018). The vulnerability and plasticity of the developing brain during infancy may explain these findings (Prado et al., 2015). These findings also indicate that children who may be at higher risk of motor development delays may benefit more from these interventions (Matias, Mridha, et al., 2017). Similarly, the *Lancet* series on Maternal and Child Under nutrition (2021) support the use of SQ‐LNS for children at risk and included it in the list of recommended interventions. Furthermore, SQ‐LNS is reported to be more cost‐effective than other interventions in reducing child mortality, preventing child malnutrition and improving child development (UNICEF, 2023).

Contextual factors, such as the initial prevalence of malnutrition, the level of household food security, the calorie content of traditional complementary foods and the accessibility of locally grown foods rich in micronutrients, may explain variations in findings across studies (Pérez‐Escamilla & Moran, 2017).

The ability of nutrition studies to find effects on child development can also be influenced by the choice of the assessment tool and the age of the assessment (Prado & Dewey, 2014). Each development assessment tool has unique psychometric properties that must be considered when analysing infant development. It is also important to validate the cultural background rather than just follow the western standards of reference (Formiga & Linhares, 2015). Systematic bias can be introduced if a test developed in a high‐income country is used in a low‐income country without adaptation (Prado & Dewey, 2014). CREDI used in the current study is designed to be culturally and linguistically neutral. Adjustments of the tool to the local contexts are usually not recommended. (McCoy et al., 2018b). As the developmental scores tend to be more stable after 2 years of age, assessments performed before the given age may not be accurate or sensitive enough to detect the effects of the intervention (Fernald et al., 2017).

In the given study, the effect of intervention was not sustained after children stopped receiving nutrient supplements. The lack of a sustained effect of nutrition intervention is also reported by other studies (Kirkwood et al., 2023; Prado, Adu‐Afarwuah, et al., 2016; Prado, Maleta, et al., 2016; Yousafzai et al., 2014). Research evidence showed that the observed benefits of an intervention may decrease over time. Decreasing effects over time, especially in high‐risk environments, may be attributable to environmental influences on children's developmental tracks after the intervention period (Fernald, et al., 2017). It is therefore important to investigate the biological and environmental factors that support or inhibit the sustained effects of early nutritional intervention (McCormick et al., 2006).

Another phenomenon referred to as ‘Latent effects’ (Maggi et al., 2010) is also observed in different studies. Sometimes the effect of early exposure to biological or environmental factors becomes manifest years and decades later, regardless of earlier experience with intervention as observed in different studies (Ocansey et al., 2019; Pollitt et al., 1993). A follow‐up study in Pakistan found higher motor development scores for children who received enhanced nutrition intervention (Yousafzai et al., 2016). In another study in Ghana significant effect was observed on socioemotional development of children at 4–6 years of age (Ocansey et al., 2019). Effects of nutrition intervention on cognitive development were not only observed immediately after the intervention period, but cognitive gains being sustained through school age and in adulthood (Ocansey et al., 2019) in a study from Guatemala, one of the longest intervention follow‐up study (Pollitt et al., 1993). The findings also suggest that intervention for longer duration may be of more benefits and may have sustained effect for longer duration.

It is suggested that developmental assessment at early childhood may convey a limited picture of the effect of nutrition interventions; particularly pertaining to those who may have smaller but clinically significant impact at population level (Larson & Biggs, 2020). Since there have been few randomized trials, and even fewer have assessed developmental outcomes in adolescence and adulthood, the most effective timing for nutritional supplementation is still not clear (Prado & Dewey, 2014). It is therefore suggested that regardless of the effect on child development, follow‐up studies for longer duration are required to investigate the long‐term effects of LNS for informed decision and policy‐making in the field of public health nutrition (Larson & Biggs, 2020).

The cluster design reduces the risk of contamination (sharing of the supplements in the participants) across clusters as they were located with clear demarcation and distances. The focus on the first 1000 days of life, a critical period for child development, was a real strength of the current study. Moreover, it is the first study in Pakistan that evaluated the effectiveness of LNS targeting that critical period. However, more studies with more robust design (randomization) and methodology are warranted. The study utilized the existing health services in terms of the hospitals and the LHWs to deliver the intervention, which is more pragmatic. The translation and cultural adaptation of CREDI (approved by the CREDI team, at Harvard University [https://credi.gse.harvard.edu/credi-translations]) was useful and added some contribution to the scientific literature for future use.

Though the allocation of participants to the study groups was non‐randomized, the groups were comparable in terms of a wide range of participant characteristics at baseline. However, non‐randomized nature of the study may raise question as whether the effect can be attributed to the intervention. In addition, without randomization, it may be difficult to establish the causality. Attrition due to loss to follow‐up is another limitation, though it was non‐differential loss. However this huge attrition was adjusted in the final analysis. In the given study the LNS has a significant effect on child development, but the effect size was small. The impact of nutrition interventions on the development of young children in LMICs has been found to be less significant (Prado, Adu‐Afarwuah, et al., 2016). Smaller effect sizes are also reported in IPD meta‐analysis by Prado et al. (2021) and in other studies as well (Larson et al., 2018; Prado, Adu‐Afarwuah, et al., 2016; Tofail et al., 2018). Another limitation is inability to blind the data collectors and participants of intervention which may have led to a systematic bias in the way the developmental interviews were conducted or interpreted by the interviewers or caregivers.

### 4.1 Policy & research implications

The research on early childhood development is reltively new in Pakistan; therefore; understanding ECD, its risk and protective factors need to be expolored in the local context. Though the current study focused on the critical period of child development, child development is an ongoing process and it is important to understand how the effect of interventions over the life course is sustained (Yousafzai et al., 2014). Supplementing lactating women with LNS does not improve child growth, but research may help in understanding the production and quality of breast milk and its micronutrient content (USAID, 2021). There is strong evidence that children in high‐risk environment (lower socioeconomic group, poor nutritional status or receiving low home stimulation) benefit more from SQ‐LNS and therefore can be one promising intervention for improving ECD (Prado et al., 2021).

Nutrition interventions alone are effective in improving ECD; however, evidence also indicates that better developmental outcomes can be achieved if nutritional interventions are combined with other components of nurturing care such as health, stimulation and early learning (Dulal et al., 2021; Prado et al., 2021; WHO, 2020). In addition, comprehensive interventions that run for longer durations have frequent contact with participants and engage the communities are suggested to be more effective in improving ECD in developing countries like Pakistan (Yousafzai et al., 2014). Engagement of LHWs for delivery of nutrition intervention has programmatic and policy implications. Evidence indicates that LHWs can provide a channel for promoting ECD. However, it is important to know that whether LHWs can effectively deliver the ECD‐related interventions without compromising their assigned tasks (Yousafzai et al., 2014). Further research is required to compare the relative effectiveness and cost‐effectiveness of commercially available nutrient supplements versus homemade energy‐dense food for pregnant women and children (Das et al., 2019).

## 5 CONCLUSION

The loss of early childhood developmental potential is a crucial issue in LMICs. The given study found a positive effect of prenatal/postnatal and children supplementation with LNS on child development who received intervention; however, the effect of intervention was not sustained after the intervention was stopped. Follow‐up studies are required to investigate the long‐term effect of nutrition intervention. More research is required to understand how nutritional and environmental factors affect the development of the brain. Additionally, more context‐specific data are required to determine the ideal composition, age group, context/setting and cost‐effectiveness.

## AUTHOR CONTRIBUTIONS

Ayesha Imtiaz and Muhammad Naseem Khan conceptualized and designed the study, performed research, did statistical analysis, and interpretation, original draft write‐up, and writing–review and editing. Zia ul Haq contributed to the critical review of the manuscript. Suhail A. R. Doi contributed to data analysis and critical review of the manuscript. Sheraz Fazid contributed to data acquisition and editing of the manuscript. The first draft of the manuscript was written by Ayesha Imtiaz and all authors commented on previous versions of the manuscript. All authors read and approved the final manuscript.

## CONFLICT OF INTEREST STATEMENT

The authors declare no conflict of interest.

## Supporting information

Supporting information.

## Data Availability

The data that support the findings of this study are available on request from the corresponding author. The data are not publicly available due to privacy or ethical restrictions.
