# Supplementary material for: Effectiveness of lipid‐based nutrient supplementation during the first 1000 days of life for early childhood development: A community‐based trial from Pakistan
Source: Matern Child Nutr. 2024 Sep 24;21(1):e13727. doi: 10.1111/mcn.13727 (PMC11650056; doi:10.1111/mcn.13727)
Supplement: Supplementary file 1 — Supporting information. [file MCN-21-e13727-s001.docx]

**Supplementary material**

**Figure A1** - Directed acyclic graph for minimally sufficient set of confounding covariates

A directed acyclic graph was used to explore the association between the exposure (Lipid based Nutrient supplementation) and outcome (child development) and to identify the potential confounding factors to inform adjustment of the regression models.

**Table A1:** Nutrient and energy contents of the Lipid-based Nutrient Supplement


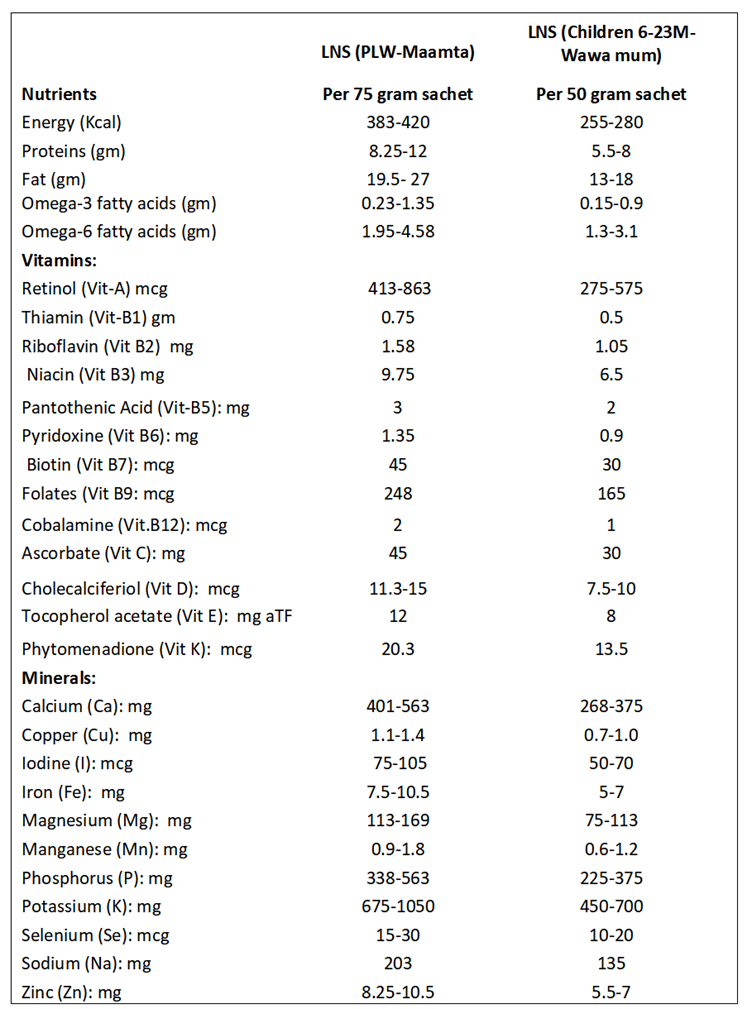


LNS was available in paste form with a sweet taste. The LNS for pregnant and lactating women (local name: Maamta) was available in pink sachet. The dose of one sachet (75 gm) per day provides the Recommended Daily Allowance (RDA) for most micronutrients and energy requirements. The LNS for children 6-23 months of age (local name: Wawa mum) was available in a yellow sachet. The daily dose of 50 grams per sachet fulfills one-fourth of daily energy requirements and most of the micronutrients according to Recommended Daily Allowance (RDA). Main ingredients of LNS included peanut/roasted chickpeas, skimmed milk powder, sugar, minerals & vitamins, vegetable oil, emulsifier, and antioxidant.

Abbreviations: Kcal, Kilo calories; mg, milligram; mcg, microgram; Vit., Vitamin

**Table A2:** Estimated intervention effects for overall and domain specific development, compared between intervention and control arms at first development assessment applying unweighted regression analysis **(n= 689)**

| **Child development scores** | **B Coef. (95% CI) P-value** | **R-Squared Link test (_hatsq)** |
| --- | --- | --- |
| **Overall**  Intervention | 0.46 (0.23, 0.68) <0.001 | 0.13 0.76 |
| **Cognitive**  Intervention | 0.29 (0.13, 0.44) <0.001 | 0.13 0.32 |
| **Motor**  Intervention | 0.47 (0.31, 0.64) <0.001 | 0.14 0.61 |
| **Language**  Intervention | 0.39 (0.22, 0.55) <0.001 | 0.20 0.33 |
| **Socioemotional**  Intervention | 0.27 (0.11 0.43) 0.001 | 0.18 0.38 |

Models adjusted for child age, gender, poverty score, household food insecurity score, mother education, mother age, father education, living structure

Comparing intervention with control group

** p < 0.01

**Table A3:** Estimated intervention effects for overall and domain specific development, compared between intervention and control arms at second development assessment applying unweighted regression analysis**(n=608)**

| **Child development scores** | **B Coef. (95% CI) P-value** | **R-Squared Link test (_hatsq)** |
| --- | --- | --- |
| **Overall**  Intervention | -0.34 (-0.52, -0.16) <0.001** | 0.15 0.62 |
| **Cognitive**  Intervention | -0.21 (-0.37, -0.06) 0.008** | 0.14 0.46 |
| **Motor**  Intervention | -0.19 (-0.37, -0.004) 0.045* | 0.14 0.56 |
| **Language**  Intervention | -0.40 (-0.57, -0.23) <0.001** | 0.17 0.42 |
| **Socioemotional**  Intervention | -0.21 (-0.37, -0.05) 0.009** | 0.15 0.14 |

Models adjusted for child age, gender, poverty score, household food insecurity score, mother education, mother age, father education, living structure

Comparing intervention with control group

*p < 0.05

** p < 0.01

The unweighted analysis is done. Results are comparable to those of weighted analysis both at first and second developmental assessment indicating that attrition did not affect the results.
